# Affibody-Binding Ligands

**DOI:** 10.3390/ijms21113769

**Published:** 2020-05-27

**Authors:** Annalisa Barozzi, R. Ashton Lavoie, Kevin N. Day, Raphael Prodromou, Stefano Menegatti

**Affiliations:** 1 Department of Chemical and Biomolecular Engineering, North Carolina State University, Raleigh, NC 27695-7905, USA; annalisa.barozzi@gsk.com (A.B.); ashton.lavoie@bridgebio.com (R.A.L.); knday@ncsu.edu (K.N.D.); rprodro@ncsu.edu (R.P.); 2 Biomanufacturing Training and Education Center (BTEC), North Carolina State University, Raleigh, NC 27695-7905, USA

**Keywords:** affibody, peptide ligands, affinity chromatography, biomanufacturing, protein purification

## Abstract

While antibodies remain established therapeutic and diagnostic tools, other protein scaffolds are emerging as effective and safer alternatives. Affibodies in particular are a new class of small proteins marketed as bio-analytic reagents. They feature tailorable binding affinity, low immunogenicity, high tissue permeation, and high expression titer in bacterial hosts. This work presents the development of affibody-binding peptides to be utilized as ligands for their purification from bacterial lysates. Affibody-binding candidates were identified by screening a peptide library simultaneously against two model affibodies (anti-immunoglobulin G (IgG) and anti-albumin) with the aim of selecting peptides targeting the conserved domain of affibodies. An ensemble of homologous sequences identified from screening was synthesized on Toyopearl^®^ resin and evaluated via binding studies to select sequences that afford high product binding and recovery. The affibody–peptide interaction was also evaluated by in silico docking, which corroborated the targeting of the conserved domain. Ligand IGKQRI was validated through purification of an anti-ErbB2 affibody from an *Escherichia coli* lysate. The values of binding capacity (~5 mg affibody per mL of resin), affinity (K_D_ ~1 μM), recovery and purity (64–71% and 86–91%), and resin lifetime (100 cycles) demonstrate that IGKQRI can be employed as ligand in affibody purification processes.

## 1. Introduction

While still dominated by monoclonal antibodies, the landscape of therapeutic and diagnostic proteins recently witnessed the emergence of other species, in particular small-molecular-weight scaffolds [1,2], like adnectins [3], anticalins [4] DARPins (designed ankyrin repeat proteins) [5], knottins [6], and affibodies [7]. Unlike antibodies, which are difficult to produce and formulate, and which suffer from low tissue permeation and potential immunogenicity due to their size and molecular complexity [8,9], small protein scaffolds can be expressed at high titer in bacteria (e.g., *Escherichia coli*), show little to no batch-to-batch variability, and possess highly tailorable binding affinity and specificity, as well as high tissue permeation ability [2].

Among small protein scaffolds, affibodies are one of the most prominent classes, with more than a dozen products on the market for analytical and research scope, as well as a growing body of literature supporting their value for therapeutic and diagnostic applications [10,11,12]. Affibodies are 58-amino-acid proteins (molecular weight of about 6.5 kDa) derived from the Z domain of staphylococcal Protein A, and they are structured as a triple α-helix bundle [13]. The native Z domain was widely commercialized as an affinity ligand for the purification of antibodies by affinity chromatography [14], and it was extensively manipulated by combinatorial engineering and in vitro selection methods to obtain protein-binding affibodies [15]. These comprise 13 surface-displayed amino acids on helices 1 and 2 that are tailored to ensure selective binding of the target protein, while helix 3 and part of helix 1 are maintained constant (Table 1) [16]. As small proteins with no post-translational modifications, affibodies can be produced efficiently in bacteria (e.g., *E. coli*), enabling affordable, high-volume production [17,18]. The ability to display a functional amino acid (e.g., cysteine) on the C- or N-terminus of affibodies without affecting their structure and binding activity facilitates their conjugation to fluorescent probes or therapeutic payloads, or onto chromatographic materials or sensor hardware [13,19].

A conspicuous body of literature is now available on the application of affibodies in the bioanalytical and medicinal fields. For example, affibodies were used as (i) alternatives to antibodies to develop cancer therapeutics (e.g., breast [20] and colorectal [21]) or ELISA kits for quantifying human plasma proteins [22], and to functionalize ProteOn^TM^ GLM sensor chips for detecting human epidermal growth factor receptor 2 (HER2)-binding affibody (ZHER2) and ZHER3 breast cancer markers [23], (ii) radiological tracers for in vivo medical imaging, owing to their lower circulation time, higher tissue permeation, and better imaging contrast as compared to antibodies [24], (iii) drug vectors, either in polyethylene glycol (PEG)-ylated or poly(phenylene sulfone) (PAS)-ylated forms to prevent fast kidney clearance, for radiometal-based therapy, or to decorate vesicles loaded with anti-cancer small interfering RNA (siRNA) [25], and finally, (iv) as ligands for the purification of blood factors [26] and antibodies [27] by affinity chromatography.

Unlike downstream manufacturing of antibodies, which relies on Protein A-based capture technology, the purification of affibodies does not yet benefit from an established platform technology. Thus, despite their therapeutic potential [28] and having received Food and Drug Administration (FDA) approval for clinical treatment [29], affibodies are available on the market in limited amounts and high price. The development of an affinity-based capture technology targeting the constant regions of affibodies in α-helix 3 and α-helix 1 holds great promise toward streamlining the manufacturing of affibodies and reducing their cost. Synthetic peptides are ideal scaffolds to develop cost-effective ligands with excellent biorecognition ability and high biochemical stability [30,31,32,33,34,35].

To identify peptide ligands with broad affibody-binding activity, we screened a solid-phase (one-bead one-peptide, OBOP) combinatorial library [36] of linear 6-mer peptides against an anti-human immunoglobulin G (hIgG) and an anti-human serum albumin (HSA) model affibodies using an orthogonal fluorescence-based selection method. The anti-hIgG and anti-HSA affibodies were labeled with either a red (AlexaFluor 488) or a green (AlexaFluor 594) fluorescent dye, and incubated simultaneously with the OBOP library pre-blocked with a mixture of host cell proteins (HCPs) obtained from a clarified *E. coli* cell lysate. After incubation, the beads were sorted into positive leads, carrying strong red and green fluorescence, and negative beads, carrying single, either red or green, or no fluorescence. The selection of beads displaying both colors at high intensity was adopted to identify peptides that bind affibodies through their constant region with high affinity and selectivity. As done in prior work [37,38], the peptides carried by the selected beads were cleaved in alkaline conditions and sequenced by liquid chromatography coupled with electrospray ionization tandem mass spectrometry (LC–ESI-MS/MS). Sixteen peptides selected based on sequence homology were synthesized on Toyopearl^®^ AF-Amino-650M and evaluated via affibody binding studies using a 1:1 solution of model affibodies in non-competitive conditions (i.e., pure affibody in phosphate-buffered saline (PBS), pH 7.4). Four sequences selected by affibody yield, namely, IGKQRI, IHQRGQ, KSAYHS, and DIRIIR, which were then evaluated in competitive conditions (i.e., affibody spiked in clarified *E. coli* cell lysate) to select a final peptide that captures affibodies selectively and releases them effectively under mild elution conditions. Providing an affibody recovery >95% and purity of 94%, peptide IGKQRI was selected as final ligand candidate, and validated against a third, anti-ErbB2 affibody. Notably, IGKQRI–Toyopearl resin was capable of purifying the anti-ErbB2 affibody from a clarified *E. coli* cell lysate with 91.5% recovery and 95.5% purity. We then measured the equilibrium binding capacity (Q_max_) and affinity (K_D,Langmuir_) of the IGKQRI–GSG–Toyopearl adsorbent via static binding experiments with pure affibodies. While the values of binding capacity were rather modest (4.86–5.31 mg of affibody per mL of resin), the values of K_D,Langmuir_ were on par with those typical of peptide ligands (~10^−6^ M). The ability of IGKQRI to target the constant region of affibodies was corroborated by binding studies in silico, by docking the structure of IGKQRI on three model affibodies published on the Protein Data Bank, namely, anti-ZHER2 (Protein Data Bank (PDB) identifier (ID): 2KZI) [39], anti-ZTaq (2B89) [40], and anti-amyloid beta A4 protein (2OTK) affibodies [41], using the docking software HADDOCK [42,43,44] in combination molecular dynamics (MD) simulations. The resulting values of K_D,in silico_ were found to be in line with the K_D,Langmuir_ data. Finally, we conducted a lifetime study of the adsorbent by performing repeated chromatographic cycles, each followed by a strong acid regeneration step, and we monitored the value of product recovery while increasing the number of injections. Over 100 chromatographic cycles, we observed a 9% decrease in yield. These results collectively indicate that the peptide IGKQRI shows promise toward being employed as a ligand for the affinity-based capture of affibodies in an industrial purification process.

## 2. Results

### 2.1. Identification of Affibody-Binding Peptides by Screening an Unbiased Library of Linear Peptides

**Figure A1:**
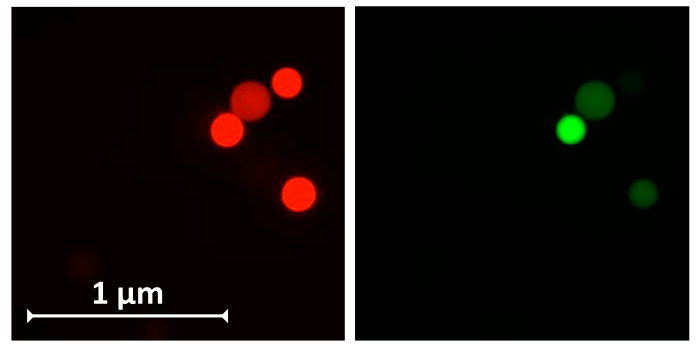
Representative fluorescence microscope images of library beads binding red- and green- labeled affibodies at different binding ratios.

**Figure A2:**
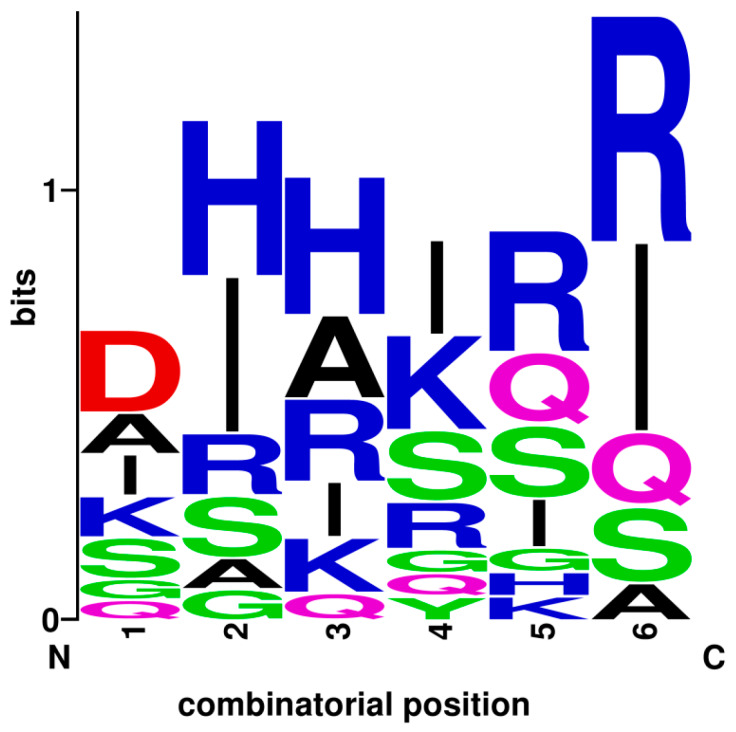
Sequence logo of combinatorial positions X_1_–X_6_ of affibody-binding peptides discovered via library screening. The figure was obtained using Weblogo (https://weblogo.berkeley.edu).

A “one-bead one-peptide” (OBOP) library of linear peptides was initially built on hydroxymethylbenzoic acid (HMBA)-ChemMatrix resin following the “split-couple-and-recombine” (SCR) method described by Lam et al. [45], and screened to discover affibody-binding peptide ligands by adapting selection methods developed by our group [37,38]. The parameters adopted for library design and screening were tailored based on the properties of the homologous regions (α-helices 1 and 2) of affibodies, as outlined in Appendix A (Table A1) and Appendix B. To impart a broad affibody-binding activity to the selected peptides, we adopted two model targets, namely, an anti-IgG [46,47] and an anti-HSA affibody [48]. These were each labeled with two fluorescent dyes, either the green AlexaFluor 488 (AF488) or the red AlexaFluor 594 (AF594), resulting in two orthogonal target pairs, namely, a green anti-IgG affibody and a red anti-HSA affibody, as well as a red anti-IgG affibody and a green anti-HSA affibody. To ensure binding selectivity, the library was screened in competitive conditions, that is, by mixing the fluorescently labeled affibodies in clarified *E. coli* cell lysate. To ensure targeting of the constant portion of affibodies, aliquots of the library were incubated with either target pair, and only the beads carrying both red and green fluorescence were chosen (Figure A1, Appendix B). The peptides carried by the selected beads were identified by liquid chromatography coupled with electrospray ionization tandem mass spectrometry (LC–ESI-MS/MS), following a method developed in prior work [37,38]. Sixteen sequences, listed in Table 1, were selected for their homology in amino-acid composition and sequence, as shown by their “sequence logo display” plot (Figure A2, Appendix B).

### 2.2. Secondary Screening: Affibody-Binding Studies in Non-Competitive Conditions

Ten peptides selected by sequence homology, namely, ARISRQ, IGKQRI, DIRIIR, QAAKRI, SHHSQR, DIHIRR, DHHKKA, DIRIQR, KSAYHS, and IHQRGQ, were tested as ligand candidates through binding studies in non-competitive conditions (pure affibody). The peptides were synthesized directly on Toyopearl AF-Amino 650 M resin by Fmoc/tBu synthesis [49]; polymethacrylate-based Toyopearl resin was chosen as a chromatographic support for secondary screening in place of ChemMatrix, owing to its chemical resistance to the reagents and solvents used for peptide synthesis, low non-specific protein binding, and mechanical strength [50]. The peptide–Toyopearl adsorbents were individually incubated with a 1:1 solution of AF594-labeled anti-HSA affibody and AF488-labeled anti-IgG affibody at the concentration of 1 mg/mL for 1 h at room temperature under gentle rotation. After washing with PBS, affibody elution (EL) was performed under acidic conditions (pH 3.8), followed by resin regeneration (R) using a harsh denaturing buffer (0.45% *w*/*v* 3-[(3-cholamidopropyl)dimethylammonio]-1-propanesulfonate (CHAPS) in 0.1 M glycine, pH 2.5). The amounts of affibodies in the unbound (UB), elution (EL), and regeneration (R) fractions, measured by fluorescence spectroscopy, were utilized to calculate the values of flow-through (FT) ratio (mass of affibody in the FT fraction vs. mass of fed antibody) and yield (mass of affibody eluted vs. mass of bound affibody), listed in Table 2. Sequences IGKQRI, IHQRGQ, KSAYHS, and DIRIIR returned the highest values of product yield and were, therefore, selected for further evaluation in competitive conditions (Section 2.3). Among them, sequences IGKQRI, IHQRGQ, and KSAYHS showed equal capture of IgG-binding and HSA-binding affibodies, which suggests targeting of the constant region of the affibody molecules, and they were selected for in silico docking studies (Section 2.5).

### 2.3. Affibody-Binding Studies in Competitive Conditions

**Figure 1:**
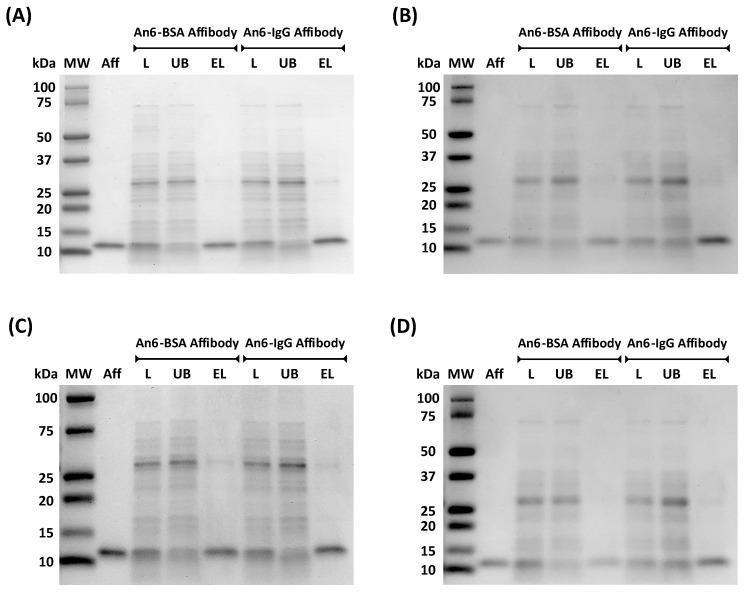
SDS-PAGE (non-reducing conditions, Coomassie stained) of unbound (UB) and elution (EL) fractions obtained by purifying anti-HSA and anti-hIgG affibodies from a clarified *Escherichia coli* cell lysate using (**A**) IGKQRI–GSG–Toyopearl, (**B**) IHQRGQ–GSG–Toyopearl, (**C**) KSAYHS–GSG–Toyopearl, and (**D**) DIRIIR–GSG–Toyopearl resins. Labels: MW, molecular weight marker; L, load; UB, unbound; EL, elution at pH 3.8; Aff, affibody standard (pure anti-HSA affibody).

The adsorbents IGKQRI–GSG–Toyopearl, IHQRGQ–GSG–Toyopearl, KSAYHS–GSG–Toyopearl, and DIRIIR–GSG–Toyopearl resins were utilized to evaluate the ability of the selected sequences to purify affibody molecules from complex sources. To this end, a feed sample was prepared by spiking fluorescently labeled anti-HSA and anti-IgG affibodies at a 1:1 molar ratio into clarified *E. coli* cell lysate, to obtain a final concentration of 0.4 mg/mL of affibody and 2 mg/mL of bacterial HCPs. The peptide–Toyopearl adsorbents were incubated with 200 μL of feed sample for 30 min at room temperature under gentle rotation. The unbound (UB) and elution (EL) fractions were analyzed by fluorescence spectroscopy and SDS-PAGE (Figure 1) to determine the values of recovery and purity for both affibody products (Table 3). It is noted that the mass of the affibody product measured electrophoretically appears to be double than the theoretical 6.5 kDa; this is likely due to dimerization of the affibodies by formation of a disulfide bond through their C-terminal cysteine residues.

**Figure 2:**
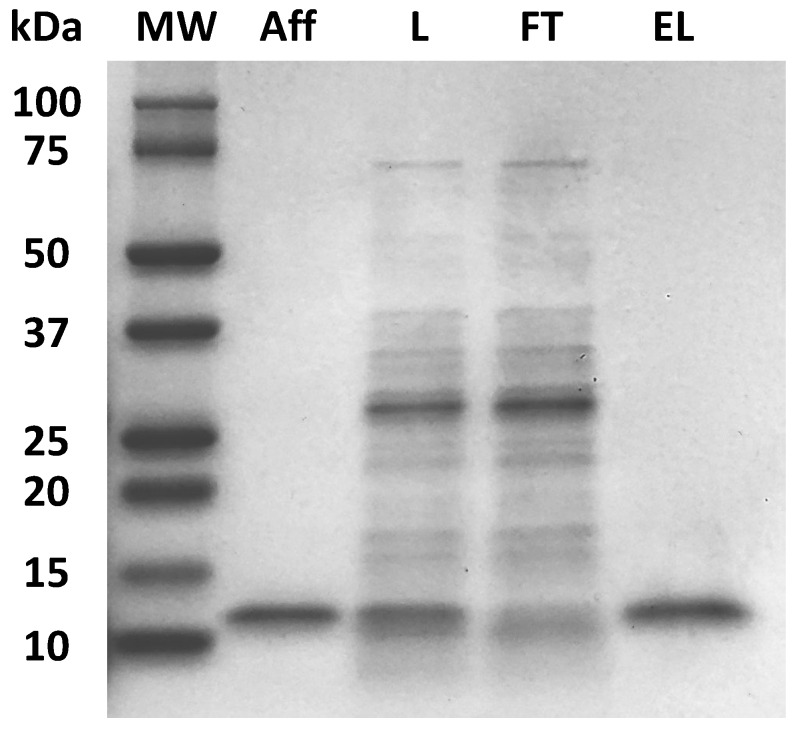
SDS-PAGE (non-reducing conditions, Coomassie staining) of flow-through (FT) and elution (EL) fractions obtained by purifying anti-ErbB2 affibody from a clarified *E. coli* cell lysate using a column packed with IGKQRI–GSG–Toyopearl resin. Labels: MW, molecular weight marker; L, load; UB, unbound; EL, elution at pH 3.8; Aff, affibody standard.

IGKQRI–GSG–Toyopearl resin was found to offer the best combination of product yield (~95% for anti-HSA affibody and ~97% for the anti-IgG affibody) and purity (94–95%), and it was, therefore, further evaluated for its ability to purify an anti-ErbB2 therapeutic affibody from a clarified *E. coli* lysate (0.2 mg/mL anti-ErbB2 affibody; 2 mg/mL *E. coli* HCPs). Sample loading onto a column packed with IGKQRI–GSG–Toyopearl resin was performed at the residence time of 2 min, followed by elution at pH 3.8 (EL) and regeneration at pH 2.5 (R). The recovery of anti-ErbB2 affibody was 91.5%, while the purity, determined by densitometry analysis of the SDS-PAGE (Figure 2) of the eluted fractions, was 95.5%, corresponding to 10-fold product enrichment. These results indicate that the adsorbent IGKQRI–GSG–Toyopearl resin possesses a broad binding ability for affibodies, and it was, therefore, selected for final evaluation in terms of binding isotherm (Section 2.4) and lifetime studies (Section 2.6).

### 2.4. Binding Isotherms of Model Affibodies on Peptide-Based Adsorbents

**Figure 3:**
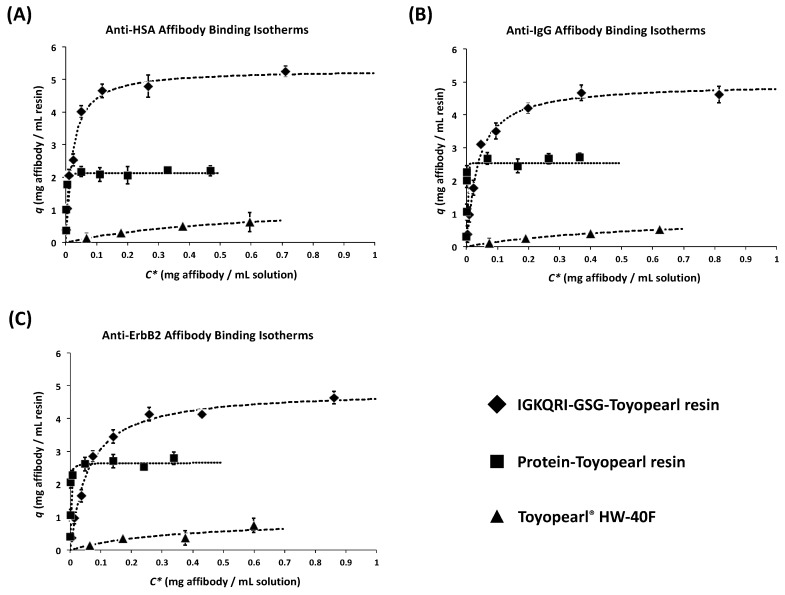
Adsorption isotherms of (**A**) anti-HSA affibody on IGKQRI–GSG–Toyopearl resin, HSA–Toyopearl, and Toyopearl^®^ HW-40F resins; (**B**) anti-IgG affibody on IGKQRI–GSG–Toyopearl resin, hIgG–Toyopearl, and Toyopearl^®^ HW-40F resins; (**C**) anti-ErbB2 affibody on IGKQRI–GSG–Toyopearl resin, ErbB2–Toyopearl, and Toyopearl^®^ HW-40F resins.

The adsorption isotherms of anti-IgG, anti-HSA, and anti-ErbB2 affibodies on IGKQRI–GSG–Toyopearl resin were performed as described in prior work [37,51]. Toyopearl resins functionalized with human IgG and serum albumin were utilized as corresponding positive controls, while Toyopearl^®^ HW-40F was utilized as a negative control. Briefly, the adsorbents were incubated with solutions of target affibody at concentrations ranging between 0.01 and 2 mg/mL until binding equilibrium was reached (2.5 h). The values of bound affibody per volume of resin (q) and the corresponding equilibrium concentration of unbound affibody (C*) were fit to a Langmuir isotherm model to determine the value of maximum binding capacity (Q_max_) and dissociation constant (K_D,Langmuir_). The adsorption isotherms of the anti-IgG, anti-HSA, and anti-ErbB2 affibodies on IGKQRI–GSG–Toyopearl resins, as well as positive and negative controls, are reported in Figure 3 A, B, and C, respectively. The corresponding values of Q_max_ and K_D,Langmuir_ are reported in Table 4. As anticipated, the peptide ligand IGKQRI was found to bind all target affibodies with comparable affinity and binding capacity. The relatively minor differences in Q_max_ and K_D,Langmuir_ among the three peptide–Toyopearl adsorbents are likely due to the small size and proximity of the constant and variable regions of affibodies, which make the variations in amino-acid sequence of the variable region affect the interaction between the peptide ligand and the constant region. The higher affinity of the positive control adsorbents, which were constructed using as ligands the proteins targeted by the model affibodies, was also anticipated; affibodies are engineered to exhibit a nanomolar affinity for the target proteins. On the other hand, the K_D,Langmuir_ of the affibody–protein interaction obtained from the adsorption isotherm studies was ~10^−7^–10^−8^ M. This is likely due to the conjugation of the protein targets on the solid phase, which limits the display of the affibody-binding sites and can alter their tertiary structure, thereby negatively impacting the binding strength. A study on the dependence of binding capacity (Q_max_) upon ligand size (peptide vs. protein ligands) and ligand density on the chromatographic resin is presented in Appendix C.

### 2.5. Computational Docking Studies of Selected Peptides

To visualize the interaction between the selected IGKQRI peptide and the target affibodies at the molecular level, we performed molecular docking studies of IGKQRI using three affibodies published on the Protein Data Bank (PDB) as model targets. While a number of crystal structures were published, none of the commercially available affibodies were modeled. Therefore, for our in silico studies, we resolved to utilize biomedically relevant affibodies, namely, a ZHER2-binding affibody (PDB ID: 2KZI), a candidate biotherapeutic alternative to monoclonal HER2-targeting antibody trastuzumab, an amyloid beta A4 protein-binding affibody (2OTK), which targets oligomers and aggregates of the amyloid-beta (Abeta) peptide found in Alzheimer’s disease, and a Protein A-binding affibody (1H0T), as model structures.

The coordinate file of the peptide IGKQRI–GSG, constructed using PyMOL [52] and equilibrated via atomistic molecular dynamics (MD), was docked against the affibody structures, performed using the docking software HADDOCK [43,44] (version 2.1) as done in prior work [37,53]. Specifically, we adopted the three-stage HADDOCK procedure comprising *(i)* rigid docking, *(ii)* in vacuo fully flexible (both ligand and the target) refinement of the rigid docking complexes, and *(iii)* water refinement of the complexes obtained from the flexible in vacuo docking. As shown in previous work [53,54], in order to constrain the orientation of the peptides resulting from their conjugation to the surface of the chromatographic resin, the residues GSG were defined as “inactive”, so that they would be oriented outward with respect to the affibody in the docked complexes. The resulting affibody–peptide complexes were clustered in structurally similar solutions, based on the carbon alpha root-mean-square deviation (C_α_ RMSD) parameter. The clusters comprising at least 10% of the total structures were analyzed via Xscore and FireDock scoring functions to select those with the predicted highest affibody-binding energy [55]. The ranking was totaled and averaged to obtain a final list of binding positions. A 100-ns MD simulation was conducted on the three top binding poses, and the trajectories in the last 10 ns were utilized to evaluate the free energy of binding ΔG_B_ and the corresponding values of K_D,in silico_ (using Equation (2) in Section 5.2.11).

**Figure 4:**
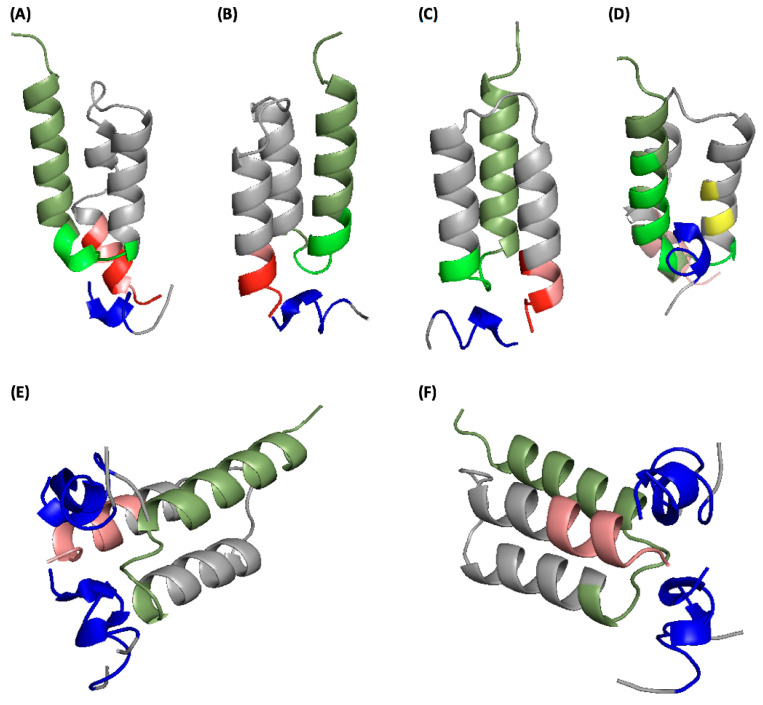
Docking of the IGKQRI–GSG peptide on (**A**) amyloid beta A4 protein-binding affibody (Protein Data Bank (PDB) ID: 2OTK); (**B**) human epidermal growth factor receptor 2 (HER2)-binding affibody (ZHER2)-binding affibody (2KZI); (**C**) ZHER2-binding affibody (90° rotation); (**D**) Protein A-binding affibody (1H0T). The IGKQRI peptide is in blue, while the GSG spacer is in gray; (**E**) different clusters of IGKQRI–GSG peptide on the ZHER2-binding affibody; (**F**) clusters of IGKQRI–GSG peptide on the ZHER2-binding affibody (180° rotation). The IGKQRI peptide is in blue, while the GSG spacer is in gray. The constant regions of the affibodies are indicated in salmon (α-helix 1), yellow (α-helix 2), and dark green (α-helix 3), as listed in Table A1 (Appendix A), while the variable regions are in gray. The portions of the constant regions of the affibodies targeted by the IGKQRI peptide are in light green (α-helix 1) and red (α-helix 3).

The structures of the affibody–peptide complexes, shown in Figure 4, indicate that peptide IGKQRI indeed targets the constant regions, namely, the α-helix 1 and α-helix 3, of the model affibody targets. In particular, the sequence DDPSQSANLL of α-helix 1, which is proximal to the variable region of α-helix 2, was targeted by IGKQRI on all affibodies (Figure 4A–C). The electrostatic interaction between the positively charged peptide ligand (net charge of +3 at pH 7.4) and the negatively charged DDPSQSANLL (net charge of −2 at pH 7.4) and the hydrogen bonding between Lys, Gln, and Arg residues of IGKQRI and the Asp, Ser, Gln, and Asn residues of DDPSQSANLL were the most relevant components of the binding free energy (ΔG_B_). Notably, the docking of peptide IGKQRI on all three affibody targets returned a number of distinct peptide clusters, all with comparable ΔG_B_ (Table 5); an example of clustered ligands on ZHER2 is shown in Figure 4 E and F. Similarly, the docking on the Protein A-binding affibody (1H0T) indicated a putative binding site of IGKQRI on some constant residues scattered on α-helix 2 (EIX_6_X_7_LPNLNX_8_). It is finally noted that the values of K_D,in silico_ obtained from the ΔG_B_ predicted in silico are in line with the values of K_D,Langmuir_ measured experimentally (Table 5), suggesting that a truly one-to-one affibody–peptide interaction occurs in the experimental binding tests.

### 2.6. Lifetime Study of IGKQRI–GSG–Toyopearl Resin

To test the ability of the peptide ligands to serve as affinity tools for affibody purification in an industrial context, we tested the ability of the peptides to yield consistent values of affibody recovery and purity through a high number of repeated chromatographic runs. The presence of a glutamine (Q) in the binding sequence excluded the possibility of using sodium hydroxide, or any other alkaline cleaning agent, for resin cleaning and sanitization. Sodium hydroxide, in fact, deamidates the carbamoylethyl group of glutamine to the corresponding carboxyethyl group of glutamic acid, thus causing a drastic change in the binding activity of the peptide ligand. Thus, strong denaturing and acid conditions (0.2 M urea in 0.25% phosphoric acid) were utilized, as done in prior work [56].

**Figure 5:**
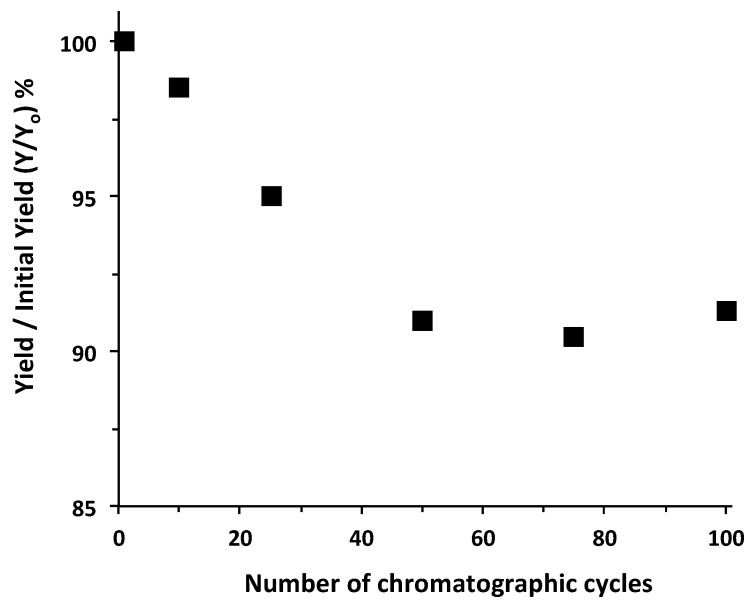
Values of yield of AF488-labeled anti-HSA affibody using IGKQRI–GSG–Toyopearl resin over 100 cycles.

Specifically, the IGKQRI–GSG–Toyopearl adsorbent was tested by performing repeated bind-and-elute cycles of AF488-labeled anti-HSA affibody. A total of 100 cycles were repeated using PBS as binding buffer, 0.2 M acetate buffer pH 3.8 as elution buffer, and 0.1 M glycine buffer added with 0.45% *w*/*v* CHAPS at pH 2.5 as regeneration and cleaning buffer. Due to the high cost of the affibody, only runs 1, 10, 25, 50, 75, and 100 were performed using the anti-HSA affibody, while all other runs were performed as blank injections. The collected eluted fractions were analyzed by fluorescence spectroscopy to determine affibody yield. The results, reported in Figure 5, clearly indicate that the peptide-based adsorbent was able to withstand multiple purification cycles without a substantial loss in binding ability. The values of affibody yield, in fact, both decreased by about 9% over 100 cycles with respect to those provided by the fresh resin.

## 3. Discussion

The purification of proteins expressed as intracellular products by bacterial systems, such as affibodies produced by *E. coli* cells, is made particularly challenging by the abundance, variety, and toxicity of the undesired intracellular species released upon cell lysis. Overcoming this challenge requires affinity ligands with optimal binding strength and selectivity. The ligands must in fact possess a balanced binding strength which enables both the capture of the target affibody, whose titer can be significantly lower than that of the other intracellular species, and its elution under mild conditions to avoid unwanted product degradation/deactivation. Furthermore, the purification of proteins that share structural and functional similarity relies on affinity ligands capable of capturing all the members of that protein family. Critical to this goal is the ability of the ligand to bind a region that is constant—or, at least, highly conserved—among all target proteins. This is showcased in the industrial purification of monoclonal antibodies, where Protein A is used as affinity ligand to capture antibodies regardless of their target antigen. Peptides are ideal candidates as ligands for such difficult bioseparations, owing to their excellent biorecognition activity, modular architecture, and chemical stability. Modern approaches to the identification of peptide ligands rely on both rational design and combinatorial screening of peptide libraries. In this vein, our study integrates in silico modeling tools with a dual-fluorescence orthogonal selection method to identify peptides that target the constant region of affibodies to serve as universal ligands for affibody purification from *E. coli* lysates. The initial sequence- and structure-based comparison between the crystal structures of affibodies available on the Protein Data Bank enabled tailoring the amino-acid composition of the peptide library using residues that favor the targeting of the constant regions of the affibodies contained in the α-helix 3 and α-helix 1. The dual-fluorescence method for library screening utilizes the sensitivity and orthogonality of fluorescence microscopy to enable the screening of ligands based on binding affinity and selectivity simultaneously. The ratio of the emission intensities (red AlexaFluor 594 vs. green AlexaFluor 488) displayed on the beads is indeed directly correlated to the ratio of bound proteins, and it is indicative of selective targeting of the constant region of affibodies in presence of *E. coli* proteins. The ability of the identified peptides to selectively capture affibodies tailored to target different proteins was confirmed both experimentally and in silico. In particular, sequence IGKQRI was conjugated to Toyopearl resin and utilized to purify anti-HSA, anti-hIgG, and anti-ErbB2 affibodies from *E. coli* cell lysates. The value of equilibrium binding capacity (Q_max_ = 4.86–5.3 g of affibody per liter of resin) is apparently lower than that characteristic of commercial affinity Protein A/G-based media for antibody purification (40–60 g/L). Upon adjusting against the molecular weight of antibodies (150 kDa) and affibodies (6 kDa), however, the resulting molar binding capacities of both media (~0.5 mmol of protein per liter of resin) are comparable. In addition, owing their small size and higher tissue penetration power, affibodies are likely to require lower therapeutic dosages compared to antibodies. These considerations indicate that values of binding capacity of IGKQRI–GSG–Toyopearl resin are in line with biomanufacturing requirements. The molecular docking and dynamic simulations of affibody–peptide interactions confirmed that IGKQRI targets the constant region of affibodies. In addition, the value of affinity (K_D_ ~1 μM) obtained from both experimental (i.e., binding isotherms) and in silico studies qualifies the peptide IGKQRI as an affinity ligand. Despite being lower than the characteristic antibody-binding strength of Protein A, the affibody–peptide affinity is sufficient to ensure good product capture in complex fluid, yet it is also quite mild to enable full recovery of bound affibodies under relatively mild conditions (pH ~4). The latter is a particularly desirable characteristic in an affinity ligand, as it reduces the risks of product degradation, denaturation, and aggregation. Of note is the ability of IGKQRI–GSG–Toyopearl resin to provide high values of recovery and purity from fluids that mimic industrial recombinant sources consistently over 100 chromatographic runs.

Collectively, these results indicate that the IGKQRI–GSG–Toyopearl adsorbent has the potential to serve as a universal adsorbent for the purification of affibodies from recombinant sources via affinity chromatography. Future work will aim to evaluate the applicability of these ligands to the purification of protein-binding affibodies from engineered *E. coli* cell lysates, as well as demonstrate their robustness towards different source fluids characterized by different profiles of HCPs and physicochemical properties (e.g., concentration, ionic strength, and pH). We anticipate that this optimization work will rely on a thorough evaluation of the properties of the chromatographic resin (particle size and pore size), ligand conjugation (peptide density and spacer arm), and loading conditions (ratio of affibody mass vs. resin volume and residence time). This work will provide opportunities to demonstrate scale-up purification of affibodies, which, despite their potential in both medical and diagnostic fields, are currently a niche product.

## 4. Conclusions

Small protein therapeutics with high biorecognition power and tissue penetration, as well as low immunogenicity potential, are poised to replace traditional monoclonal antibodies in treating solid cancer and neurodegenerative disorders, or in developing bioassays. Affibodies are among the small-scaffold proteins that show the highest translational potential in therapy and diagnostics. With the increasing number of pre-clinical and clinical studies, however, a crucial question lays on the horizon, concerning how to affordably manufacture the volume of highly purified affibodies needed to meet the demand by clinics and biotech companies worldwide. In this regard, the expression of affibodies in recombinant systems—whether bacterial or yeast—was substantially explored and optimized. Affibody purification, on the other hand, relies on affinity chromatography using tags or the protein targeted by the affibody as ligands, which are unfeasible for the large-scale manufacturing of therapeutics. The FDA, in fact, discourages the use of affinity tags, and the use of protein targets as ligands is incompatible with the goal of a platform approach to affibody purification. To address this challenge, we developed the first known affibody-binding peptide ligands using an approach integrating combinatorial screening with experimental and in silico evaluation of the affibody–peptide biorecognition events. Among the identified sequences, a selected peptide fulfills the requirements asked of affinity ligands, namely, the binding capacity, the robustness to different affibody targets, the selectivity against protein impurities, and the durability to secure long adsorbent lifetime. This study, therefore, represents the first effort toward the development of an affinity-based technology that is truly tailored to the large-scale purification of affibody-based and affibody-fused therapeutics.

## 5. Materials and Methods

### 5.1. Materials

Anti-hIgG, anti-HSA, and anti-ErbB2 affibodies were obtained from Abcam (Cambridge, MA, USA). AlexaFluor 488 (AF488, ThermoFisher Scientific, Waltham, MA, USA) and AlexaFluor 594 (AF594, ThermoFisher Scientific, Waltham, MA, USA), acetic acid glacial, sodium acetate, sodium chloride, glycine, 30% (*v*/*v*) aqueous hydrochloric acid, 85% (*v*/*v*) phosphoric acid, *N*,*N*’-dimethylformamide (DMF, MilliporeSigma, Burlington, MA, USA), dichloromethane (DCM, MilliporeSigma, Burlington, MA, USA), HPLC-grade acetonitrile, Coomassie Plus Bradford assay kit, and the Micro bicinchoninic acid assay (BCA) Protein Assay Kit were sourced from ThermoFisher Scientific (Waltham, MA, USA). Furthermore, 3-kDa molecular weight cutoff (MWCO) Amicon Ultra centrifugal filters were purchased from EMD Millipore (Burlington, MA, USA). Fmoc-protected amino acids and 2-(7-aza-1*H*-benzotriazol-1-yl)-1,1,3,3-tetramethyluronium hexafluorophosphate (HATU) were purchased from ChemImpex Inc. (Wood Dale, IL, USA). HMBA-ChemMatrix (HMBA: hydroxymethylbenzoic acid) resin was obtained from PCAS Biomatrix Inc. (Saint-Jean-sur-Richelieu, QC, Canada). Acetic anhydride, diisopropylethylamine (DIPEA), ethanedithiol (EDT), piperidine, trifluoroacetic acid (TFA), triisopropylsilane (TIPS), Tween-20, phosphate-buffered saline (PBS) pH 7.4, and a Kaiser test kit were obtained from MilliporeSigma (Burlington, MA, USA). Toyopearl AF-Amino-650 M and Toyopearl^®^ HW-40F resins were a kind gift from Tosoh Bioscience (King of Prussia, PA. Microbore polyether ether ketone (PEEK) columns 30 mm long × 2.1 mm inner diameter (I.D.) were sourced from VICI Precision Sampling (Baton Rouge, LA, USA). The *E. coli* cell lysate was donated by the Rao group (Chemical and Biomolecular Engineering, NCSU, Raleigh, NC, USA).

### 5.2. Methods

#### 5.2.1. Synthesis of Peptide Library

The hexamer library of linear peptides X_1_–X_2_–X_3_–X_4_–X_5_–X_6_ was synthesized on HMBA-ChemMatrix resin (particle diamerer of 75–150 μm, functional density of 0.8 mmol HMBA per g resin) pre-loaded with the peptide spacer GSG (G: glycine, S: serine). The peptides were synthesized via conventional Fmoc/tBu chemistry using a Syro I peptide synthesizer (Biotage, Uppsala, Sweden). Briefly, every residue (X_i_) was conjugated by performing two 15-min amino-acid couplings at 45 °C, using 3 equivalents (eq., compared to the functional density of the HMBA-ChemMatrix resin) of amino acid, 3 eq. of HATU, and 6 eq. of DIPEA in 5 mL of anhydrous DMF. The completion of each conjugation reaction was monitored after each amino acid by Kaiser test. The deprotection of Fmoc protecting groups was performed by rinsing the resin twice with 5 mL of 20% piperidine in DMF for 20 min at room temperature. The combinatorial positions X_1_–X_6_ were produced via the “split-couple-and-recombine” (SCR) technique using 10 protected amino acids, namelym Fmoc-Ala-OH, Fmoc-Asp(OtBu)-OH, Fmoc-Arg(Pbf)-OH, Fmoc-Gln(Trt)-OH, Fmoc-Gly-OH, Fmoc-His(Trt)-OH, Fmoc-Ile-OH, Fmoc-Lys(Boc)-OH, Fmoc-Ser(tBu)-OH, and Fmoc-Tyr(tBu)-OH. Briefly, *(i)* the resin was divided into 10 aliquots and each was placed in a reaction vessel (~0.17 g resin per vessel); *(ii)* after an amino-acid conjugation and removal of the Fmoc protecting group, the aliquots were combined, mixed, and re-divided. The SCR procedure was performed six times to generate the corresponding six combinatorial positions for a total of 10^6^ peptide combinations. The side chain-protecting groups were removed via acidolysis, by incubating the peptide–ChemMatrix library with a cleavage cocktail comprising TFA/TIPS/anisole/EDT (94/3/2/1) for 2 h. The resins were rinsed in DCM and DMF and stored at 4 °C.

#### 5.2.2. Conjugation of Fluorescent Dyes to Affibody Molecules

Anti-IgG and Anti-HSA affibodies were labeled with either AF488 or AF594 dye, both in thiol-reactive maleimide form, for a total of four fluorescently labeled affibodies. The affibody dimers were firstly treated with 1 mM ethylenediaminetetraacetic acid (EDTA) and 1 mM dithiothreitol (DTT) in Tris-HCl at pH 8.0 to break the disulfide bonds, and then diafiltered against 1 mM EDTA in Tris-HCl at pH 8.0 using 3-kDa MWCO Amicon Ultra Centrifugal Filters (EMD Millipore) to maintain them as thiol-free monomers in solution. Each dye was initially dissolved in anhydrous dimethyl sulfoxide (DMSO) to a concentration of 10 mg/mL, and slowly added to 100 μL of affibody solution at 2 mg/mL in Tris-HCl at pH 8.0. The reaction was allowed to proceed for 90 min at 4 °C, and then quenched with 20 μL of 0.05 M Tris-buffered saline at pH 8.0, followed by incubation at 4 °C for 1 h. The labeled affibodies were then diafiltered against PBS at pH 7.4.

#### 5.2.3. Screening of Peptide Library against Labeled Affibodies

Two affibody solutions were initially formulated; screening mixture 1 (SM1) was prepared by spiking AF488-labeled (green) anti-HSA affibody and AF594-labeled (red) anti-IgG affibody, both at 1 mg/mL, in the clarified *E. coli* cell lysate, while screening mixture 2 (SM2) was prepared by spiking AF594-labeled anti-HSA affibody and AF488-labeled anti-IgG affibody, both at a concentration of 0.5 mg/mL, in the *E. coli* lysate. The total concentration of *E. coli* was 2 mg/mL. Two aliquots of 0.1 mL of library beads were initially equilibrated with 0.1% Tween-20 in PBS at pH 7.4, and then blocked with a mixture of clarified *E. coli* cell lysate. Each aliquot was then rinsed with PBS and incubated overnight at 4 °C with either SM1 or SM2. The beads were thoroughly washed with PBS and 0.1% Tween-20 in PBS at pH 6, as done in prior work [37,38], before being isolated into 96-well polystyrene plates and individually imaged with an EVOS FL Auto Imaging System (ThermoFisher Scientific, Waltham, MA, USA). The beads carrying high green and red fluorescence were isolated and treated by multiple 1-h incubations in 0.2 M acetate buffer at pH 3.8 at room temperature and under gentle rotation to remove all bound proteins. Finally, the beads were thoroughly rinsed with deionized water and stored in acetonitrile.

#### 5.2.4. Sequencing of Selected Peptide Leads

The library beads selected as described in Section 2.4 were individually placed in 20 μL of 38 mM NaOH in 10% acetonitrile at 4 °C for 30 min to cleave the peptides carried thereon. Immediately after cleavage, the peptide solutions were pH-neutralized by adding 20–30 μL of 100 mM citrate, 10% acetonitrile, 0.1% formic acid at pH 2.9, resulting in a total sample volume of 40–50 μL. Since an injection volume of 5 μL is required for peptide sequencing by mass spectrometry, this protocol enables multiples sequencing attempts, increasing the probability of successful identification. Following pH neutralization, peptide solutions were filtered using 0.45-μm centrifugal filters by centrifugation to separate the peptide solution from the beads and any solid debris, and they were eventually dried in a centrifugal evaporator (Thermo Savant SC110 SpeedVac Vacuum Concentrator). The cleaved peptides were then sequenced by liquid chromatography coupled with electrospray ionization tandem mass spectrometry (LC–ESI-MS/MS) using a Thermo Fisher Q Exactive^TM^ High-Field Hybrid Quadrupole-Orbitrap^TM^ Mass Spectrometer coupled to an Easy LC 1200 system with an ESI (electrospray ionization) source. Liquid chromatography was performed using a Phenomenex C18 stationary phase (2.6 μm bead diameter size and 100 A pore size) packed in a New Object PicoFrit Emitter column (11.5 cm height, 75 μm I.D., 360 μm outer diameter (O.D.)). Prior to injection, the dried cleaved peptides were reconstituted in 20 μL of aqueous 0.1% formic acid solution. A volume of 5 μL of sample was injected onto the chromatographic bed, washed with a 2% acetonitrile, 0.1% formic acid buffer, and eluted using a linear gradient of acetonitrile in 0.1% formic acid elution buffer, from 2% to 80% over 1 h. The orbitrap was operated as follows: positive ion mode, acquisition full scan (*m*/*z* 400–1990) with 120,000 resolving power, MS/MS acquisition using a top N data-dependent acquisition (DDA) implementing higher-energy collisional dissociation (HCD) with a normalized collision energy (NCE) setting of 27%. Dynamic exclusion was utilized to maximize depth of proteome coverage by minimizing re-interrogation of previously sampled precursor ions. Real-time lock mass correction using the polydimethylcyclosiloxane ion at *m*/*z* 445.120025 was utilized to minimize precursor and product ion mass measurement errors. The peptide sequences were obtained by searching the acquired MS data against a peptide database in FASTA format. The database was constructed to contain all 10^6^ sequences in the peptide library based on the degenerate amino-acid combinations. The raw MS/MS data were processed using Proteome Discoverer 1.4 (ThermoFisher Scientific, Waltham, MA, USA). Searching was performed with a 5-ppm precursor mass tolerance and 0.02-Da fragment tolerance. Specified modifications included Asn and Gln deamidation, which could arise from alkaline degradation during peptide cleavage. Identifications were filtered to a strict protein false discovery rate (FDR) of 1% and relaxed FDR of 5% using the Percolator node in Proteome Discoverer.

#### 5.2.5. Affibody Binding and Elution Studies in Non-Competitive Conditions

The identified sequences were synthesized on Toyopearl AF-Amino 650 M resin by Fmoc/tBu synthesis to be tested for affibody binding in non-competitive mode. Aliquots of 50 μL of each peptide–Toyopearl adsorbent were transferred in a PCR tube, swollen in 20% methanol overnight, and copiously washed in 20% methanol to remove any residual chemical from peptide synthesis, before equilibrating in Milli-Q water followed by PBS, pH 7.4. Next, 100 μL of 50:50 solution of fluorescently labeled anti-HSA and anti-IgG affibodies (Section 2.3) at a total 1 mg/mL affibody concentration were incubated with every aliquot of peptide–Toyopearl resin for 1 h at room temperature under gentle rotation. Following incubation, the resins were pelleted by centrifugation, and the supernatant was collected and marked as “unbound” (UB) fraction. The resins were then washed with PBS and the supernatant were combined with the UB fractions. Protein elution (EL) was then performed by incubating the resins with 0.2 M acetate buffer at pH 3.8 (0.2 M acetic acid and 0.2 M sodium acetate at a 7:3 ratio) for 30 min at room temperature, and then by washing with the same elution buffer. Regeneration (R) was then performed by incubating the adsorbents with 100 mM glycine buffer at pH 2.5 added with 0.45% *w*/*v* CHAPS, at 4 °C for overnight. The collected elution fractions (EL and R) were finally equilibrated to neutral pH using PBS, pH 7.4. Finally, both UB and E fractions were diluted in PBS and analyzed by fluorescence spectroscopy.

#### 5.2.6. Affibody Binding and Elution Studies in Competitive Conditions

Three sequences selected in Section 2.6 were then evaluated for affibody binding in competitive mode. Aliquots of 100 μL of the peptide–Toyopearl adsorbents were swollen and equilibrated as described in Section 2.6. The feed sample was prepared by combining 100 μL of equimolar solution of fluorescently labeled anti-HSA and anti-IgG affibodies at a total 2 mg/mL affibody concentration with 400 μL of clarified *E. coli* cell lysate at the adjusted concentration of host cell proteins (HCPs) of 2.5 mg/mL, to obtain a final concentration of 0.4 mg/mL of affibody and 2 mg/mL of HCPs. Next, 200 μL of feed samples were incubated with each aliquot of wet peptide–Toyopearl resin for 2 h at room temperature under gentle rotation. After incubation, unbound (UB) and elution (EL) fractions were obtained as described in Section 5.2.5; they were then analyzed by fluorescence spectroscopy to determine the yield of both affibodies and finally by SDS-PAGE to determine the total purity of the eluted affibody products.

#### 5.2.7. Purification of Anti-ErbB2 Affibody Using Peptide Ligand IGKQRI

Anti-ErbB2 affibody labeled with green-fluorescent AF488 was prepared as described in Section 2.3. Next, 100 μL of the IGKQRI–Toyopearl resin was wet-packed in Microbore PEEK columns, mounted on a Waters Alliance^®^ HPLC System, and equilibrated in PBS, pH 7.4. The feed sample was prepared by spiking 100 μL of AF488-labeled anti-ErbB2 affibody at 2 mg/mL into 400 μL of clarified *E. coli* cell lysate at the adjusted HCP concentration of 2 mg/mL, to obtain a final concentration of 0.4 mg/mL of affibody and 2 mg/mL of HCPs. Next, 250 μL of feed sample was loaded on the column at a flow rate of 0.05 mL/min, corresponding to a residence time of 2 min. The resin was washed with five column volumes (CVs) of PBS, and elution was then performed with 10 CVs of 0.1 M acetate buffer, pH 3.8. Finally, the adsorbent was regenerated with 10 CVs of 0.45% *w*/*v* CHAPS in 0.1 M glycine HCl at pH 2.5, equilibrated with PBS, rinsed with water, and stored in aqueous 20% *v*/*v* methanol. All chromatographic steps were performed at a linear velocity of 0.25 mL/min (residence time of 0.4 min), and the effluent was monitored by ultraviolet (UV) spectrophotometry at 280 nm. The chromatographic fractions (UB, W, EL, and R) were analyzed by fluorescence spectroscopy and SDS-PAGE to determine the yield and purity of the anti-ErbB2 affibody.

#### 5.2.8. Analysis of the Chromatographic Fractions by Sodium Dodecyl Sulfate Polyacrylamide Gel Electrophoresis

The collected fractions were desalted into phosphate buffer at pH 7.4 using Amicon Ultra 0.5-mL centrifugal filters (3 kDa MWCO). Based on the total protein concentration determined by Bradford assay, the collected fractions were adjusted at a total protein concentration of 0.1 mg/mL, diluted 1:1 with 2× Laemmli sample buffer containing 5% β-mercaptoethanol, and incubated at 100 °C for 5 min. Next, 20 µL of each sample was loaded onto each well of a 12% Mini PROTEAN^®^ TGX precast electrophoresis gel. Then, 10 µL of Precision Plus Protein TM Dual Color Standards diluted 100× in Laemmli sample buffer was loaded in the first and last wells. Gels were electrophoresed at 100 V constant for 95 min using a Mini PROTEAN Tetra Cell (Bio-Rad, Hercules, CA, USA) linked to a PowerPac 300 power supply (Bio-Rad). Gels were fixed in 10% acetic acid, 40% ethanol in MilliQ water for 1 h. Fixed gels were washed with MilliQ water and Coomassie stained. Imaging and densitometric analysis were performed with a GelDoc XR+ (Bio-Rad) and Image Lab software (Bio-Rad) with band intensities scaled to the intensity of an affibody standard.

#### 5.2.9. Binding Isotherm of Model Affibodies on Peptide-Based Adsorbents

Eight 50-µL aliquots of IGKQRI–GSG–Toyopearl resin were initially equilibrated in PBS at pH 7.4. Eight 0.2-mL solutions of anti-hIgG, anti-hHSA, and anti-ErbB2 affibodies in PBS at concentrations ranging from 0.01 to 2 mg/mL were prepared and incubated with the aliquots of IGKQRI–GSG–Toyopearl resin for 2.5 h at room temperature under mild shaking. After separating the supernatant by centrifugation, the resin aliquots were washed. The supernatant and washing solutions were combined and analyzed by fluorescence spectroscopy to determine the equilibrium concentration of affibody solution. The mass of affibody bound by the resin was determined by mass balance. The values of affibody bound per volume of resin (q) and the corresponding values of equilibrium concentration in solution (C*) were fit to a Langmuir isotherm model (Equation (1)).

(1) $$q=\frac{Q_{\max}C^{*}}{K_{D,\mathrm{Langmuir}}+C^{*}},$$

where Q_max_ is the maximum binding capacity (mg affibody per mL resin), and K_D,Langmuir_ is the dissociation constant (µM).

#### 5.2.10. Lifetime Study of Peptide-Based Adsorbents

The binding of anti-HSA affibody was repeated on IGKQRI–GSG–Toyopearl resin 100 times, by applying the chromatographic protocol described in Section 5.2.5. Only runs 1, 10, 25, 50, 75, and 100 were performed using the anti-HSA affibody, while all other runs were performed as blank injections. The flow-through and elution chromatographic fractions were analyzed by UV spectroscopy at 280 nm to determine the dependence of affibody yield with the number of uses.

#### 5.2.11. Computational Docking Studies

Selected sequence IGKQRI was docked in silico against different model affibodies to evaluate its binding site and strength [57]. Following the procedure established in prior work [37,53,58], the coordinate files of the linear peptides IGKQRIGSG were initially designed using Pymol’s build function, and equilibrated by molecular dynamics (MD) simulation in the AMBER16 package using the AMBER ff14SB force-field parameter set [59]. The peptide was placed in a simulation box with periodic boundary conditions containing 600 water molecules (transferable intermolecular potential with three points (TIP3P) water model) [60,61,62]. The solvated system was minimized by running 10,000 steps of steepest gradient descent, heated to 300 K in a constant number, volume, and temperature (NVT) ensemble for 250 ps (1 fs time steps), and equilibrated to 1 atm in a constant number, pressure, and temperature (NPT) ensemble for 500 ps (2 fs time steps). The production run was performed in the NPT ensemble, at constant T = 300 K using the Nosé–Hoover thermostat [63,64,65] and P = 1 atm using the Parrinello–Rahman barostat [66,67]. The leap-frog algorithm was used to integrate the equations of motion and all of the covalent bonds were constrained by means of the linear constraint solver (LINCS) algorithm [68]. The short-range electrostatic and Lennard–Jones interactions were calculated with cutoffs of 1.0 nm and 1.2 nm, respectively, while the long-range electrostatic interactions were evaluated using the particle mesh Ewald method [69,70]. The atomic coordinates were saved every 2 ps, and the non-bonded interaction pair list was updated every 5 fs (cutoff of 1.2 nm). The resulting peptide structure was docked against an anti-ZHER2 affibody (PDB ID: 2KZI), an anti-ZTaq affibody (2B89), and an anti-amyloid beta A4 protein affibody (2OTK) using the docking software HADDOCK (High Ambiguity Driven Protein–Protein Docking V.2.1) [43,44]. Default HADDOCK parameters (e.g., temperatures for heating/cooling steps, and the number of MD sets per stage) were used in a “blind docking” procedure. All the residues on each affibody target with solvent accessibility of 50% or greater were defined as “active” (directly involved in the interaction between the peptide ligand and the protein), whereas all other residues were defined as “passive” (involved in the interaction as a result of the “active” residue binding). Similarly, all variable amino-acid positions on the peptide ligands were denoted as “active” while the GSG (Gly–Ser–Gly) spacer was defined as not being involved in the interaction to account for the directionality of binding. To simulate the orientation that the peptide assumes when conjugated onto a chromatographic support, in fact, the GSG trimer was constrained to be non-interacting to any of the target affibodies [71]. Docking proceeded through a three-stage protocol: (1) rigid, (2) semi-flexible, and (3) water-refined fully flexible docking. A total of 1000, 200, and 200 structures were calculated at each stage, respectively. The final structures were grouped using a minimum cluster size of 20 with a Cα RMSD < 0.5 nm using ProFit (http://www.bioinf.org.uk/software/profit/). The clusters identified for each affibody–peptide complex were scored using FireDock and XScore [55]. FireDock is an efficient method re-scoring of protein–protein docking solutions. Xscore computes the dissociation of a protein–ligand complex using an empirical equation that considers energetic factors in a protein–ligand binding process. The top three binding poses selected by FireDock and XScore were finally evaluated by 100-ns MD simulations in explicit-solvent conditions; the values of free energy of binding (ΔG_b_) were evaluated using the last 10 ns of MD trajectories, and the corresponding affinity (K_D,*in silico*_) was calculated using Equation (2).

(2) $$K_{D,in\;silico}=\frac{{\Delta G}_{b}}{\mathrm{RT}},$$

where R is the ideal gas constant, and T is the temperature in K.

## Figures and Tables

**Table 1:** Sequences of 16 affibody-binding linear peptides discovered via library screening. The “X” denotes an amino acid that could not be assigned via MS/MS sequencing. ID—identifier.

Peptide ID	Sequence	Peptide ID	Sequence
ABP1	XHHKSI	ABP9	DHHKKA
ABP2	ARISRQ	ABP10	SHHSQR
ABP3	KIIISR	ABP11	DIRIQR
ABP4	IGKQRI	ABP12	KSAYHS
ABP5	DIRRII	ABP13	XRAGRI
ABP6	DIRIIR	ABP14	IHQRGQ
ABP7	QAAKRI	ABP15	DIHIRR
ABP8	SHHSQR	ABP16	GSKKSS

**Table 2:** Sequence flow-through elution mass ratio for both anti-human serum albumin (HSA) and anti-immunoglobulin G (IgG) affibodies.

Sequence	m_FT_/m_E_ (Anti-HSA)	m_FT_/m_E_ (Anti-IgG)	Yield
DIHIRR	0.473	1.277	40.4%
DIRIIR	1.553	0.707	57.2%
SHHSQR	1.387	1.238	44.3%
QAAKRI	0.801	2.245	35%
DHHKKA	2.039	1.256	38.5%
IHQRGQ	0.781	0.446	60.3%
IGKQRI	0.628	0.448	64.7%
DIRIQR	2.401	0.445	39.5%
ARISRQ	1.709	0.885	51.7%
KSAYHS	0.796	0.405	60.1%

**Table 3:** Recovery and purity of anti-HSA and anti-IgG affibodies purified from clarified *E. coli* cell lysate using the adsorbent IGKQRI–GSG–Toyopearl, IHQRGQ–GSG–Toyopearl, KSAYHS–GSG–Toyopearl, and DIRIIR–GSG–Toyopearl resins. The values of yield were calculated as the ratio of the amount of fluorescently labeled affibody present in the eluted fractions (measured via fluorescence spectroscopy) vs. the loaded amount, whereas the values of purity were calculated by densitometric analysis of the elution (EL) lanes in the gel reported in Figure 1 using Image Lab software (Bio Rad).

Sequence	Anti-HSA Affibody		Anti-IgG Affibody	
	Recovery	Purity	Recovery	Purity
IGKQRI	95.2% ± 4.6%	94.1%	97.2% ± 1.5%	95.8%
IHQRGQ	96.1% ± 2.4%	92.6%	95.3% ± 4.5%	93.1%
KSAYHS	89.5% ± 0.7%	96.3%	88.6% ± 3.4%	96.4%
DIRIIR	74.9% ± 3.6%	96.2%	78.3% ± 2.5%	96.5%

**Table 4:** Fitting of isotherm data to the Langmuir adsorption model (TP: Toyopearl resin).

Sequence	Anti-HSA Affibody		Anti-IgG Affibody		anti-ErbB2 Affibody	
	K_D,Langmuir_ (M)	Q_max_ (mg/mL)	K_D,Langmuir_ (M)	Q_max_ (mg/mL)	K_D,Langmuir_ (M)	Q_max_ (mg/mL)
IGKQRI–GSG–TP	3.1 × 10^−6^	5.31	5.38 × 10^−6^	4.95	8.92 × 10^−6^	4.86
Protein–TP	4.9 × 10^−8^	2.13	1.8 × 10^−8^	2.54	1.15 × 10^−7^	2.56
TP HW-40F	9.2 × 10^−4^	0.85	1.0 × 10^−4^	0.93	1.4 × 10^−4^	0.56

**Table 5:** Values of binding affinity (K_D,in silico_, calculated using Equation (2) from molecular dynamics (MD)-derived values of ΔG_B_, Section 5.2.11) of the top 3 affibody–IGKQRI clusters obtained by docking the peptide IGKQRI on amyloid beta A4 protein-binding affibody, ZHER2-binding affibody, and Protein A-binding affibody, followed by MD simulation of the affibody–IGKQRI complexes in the selected poses.

Affibody–IGKQRI Top Cluster	Amyloid beta A4-Binding Affibody (PDB ID: 2OTK)	ZHER2-Binding Affibody (2KZI)	Protein A-Binding Affibody (1H0T)
1	1.9 × 10^−5^ M	7.0 × 10^−6^ M	5.8 × 10^−6^ M
2	2.1 × 10^−5^ M	1.2 × 10^−5^ M	4.1 × 10^−5^ M
3	1.4 × 10^−5^ M	8.2 × 10^−5^ M	7.2 × 10^−6^ M

**Figure A3:**
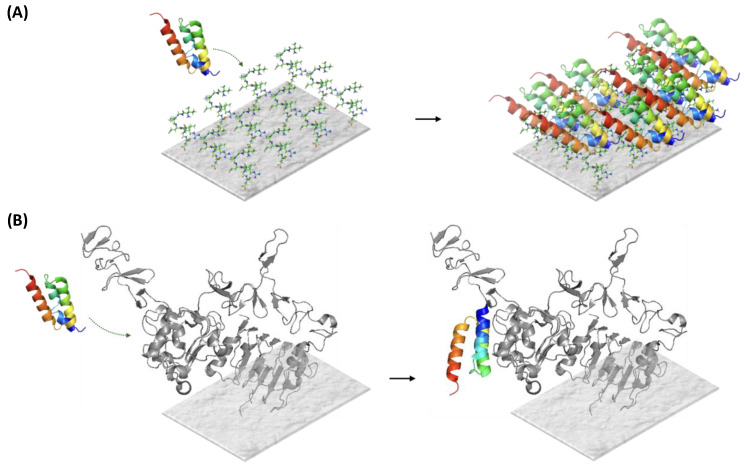
Representation of affibody binding on (**A**) IGKQRI–GSG–Toyopearl vs. (**B**) erbB2–Toyopearl resins. The peptide ligands, target affibody, and protein–affibody complex are in 1:1 molecular scale to each other.

**Figure A4:**
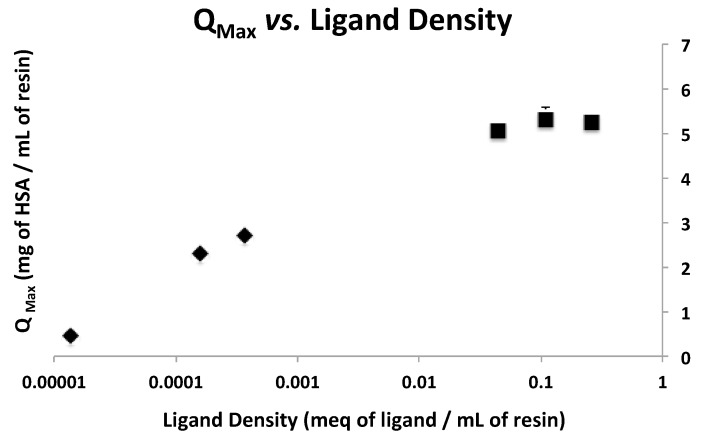
Correlation between equilibrium binding capacity of anti-HSA affibody onto (■) IGKQRI–GSG–Toyopearl resin and (♦) HSA–Toyopearl resin. Binding studies were performed in triplicate.

## Abbreviations

HCP	Host cell protein
*E. coli*	*Escherichia coli*
AF	Alexafluor
SM	Screening mixture
TP	Toyopearl resin
FT	Flow-through
UB	Unbound
W	Wash
EL	Elution
R	Regeneration
CV	Column volume
ELISA	Enzyme-linked immunosandwich assay
PBS	Phosphate-buffered saline
HMBA	Hydroxymethylbenzoic acid
SPPS	Solid-phase peptide synthesis
LC/ESI/MS/MS	Liquid chromatography coupled with tandem electrospray ionization mass spectrometry
OBOP	One-bead one-peptide
DMF	*N*’,*N*’-Dimethylformamide
DMSO	Dimethylsulfoxide
Fmoc	Fluorenylmethyloxycarbonyl
HATU	7-Azabenzotriazol-1-yloxy)tripyrrolidino-phosphonium hexafluorophosphate
DIPEA	Diisopropylethylamine
TFA	Trifluoroacetic acid
EDT	Ethanedithiol
TIPS	Triisopropylsilane
hIgG	Human immunoglobulin G
HSA	Human serum albumin
Q-ToF	Quadrupole time-of-flight mass spectrometer
HCD	High-energy collisional dissociation
MW	Molecular weight
Qmax	Maximum binding capacity
K_D_	Dissociation constant
PDB	Protein Data Bank
CHAPS	3-[(3-Cholamidopropyl)dimethylammonio]-1-propanesulfonate
SDS-PAGE	Sodium dodecylsulfate polyacrylamide gel electrophoresis
HADDOCK	High Ambiguity Driven biomolecular Docking
MD	Molecular dynamics
RMSD	Root-mean-square deviation of atomic positions
BCA	Bicinchoninic acid assay

## Appendix A. Design of the Unbiased Combinatorial Linear Peptide Library

A “one-bead one-peptide” (OBOP) combinatorial library of linear peptides was built following the “split-couple-and-recombine” (SCR) method described by Lam et al. [45] and previously by our group [37,38]. The design parameters (composition and structure) of the peptide library, namely, the choice of amino acids used in each coupling cycle and the number of coupling cycles determining peptide length, were tailored based on the properties of the homologous regions in α-helices 1 and 2 of affibodies. We resolved to construct a hexameric linear library X_1_X_2_X_3_X_4_X_5_X_6_GSG, where (i) X_1_X_2_X_3_X_4_X_5_X_6_ represents the variable segment of the peptide comprising six combinatorial positions X_i_ randomized using 10 amino acids, for a total of up to 10^6^ unique peptide sequences, and (ii) the GSG (Gly–Ser–Gly) spacer promotes the display of the variable segment of the peptide onto the solid phase, and aids in post-screening cleavage and sequencing of the selected peptides. The length of six amino acids was chosen as it offers a good balance of binding strength and selectivity and cost. The amino-acid composition of the library (Ala, Asp, Tyr, His, Lys, Ile, Gln, Arg, Gly, and Ser) was selected based on the biophysical characteristics of the constant domains of anti-ZHER2, anti-ZTaq, and anti-Amyloid beta A4 protein (2OTK) affibodies, whose amino-acid sequences are published on the Protein Data Bank (PDB IDs: 2KZI, 2B89, and 2OTK). The sequences, as well as their calculated isoelectric point (IP) and grand average hydroxypathy index (GRAVY [72]), are reported in Table A1. As the constant regions on α-helix 1 and α-helix 3 are of opposite charge, both positively (Lys and Arg) and negatively (Asp) charged amino acids were included. Furthermore, given the hydrophilicity of both regions (GRAVY ~−1) and the abundance of hydrogen bond-forming residues, electrically neutral amino acids capable of hydrogen bonding (Gln and Ser) were also included, and hydrophobic amino acids were limited to Ile and Tyr.

**Table A1:** Amino acid sequence of affibodies, where X_1_–X_13_ indicate the randomized positions, as well as the isoelectric point, net charge at pH 7, and grand average hydroxypathy index (GRAVY) index of the constant regions. Note: to mimic its connection to the rest of the affibody molecule, the C-terminus of sequence * was amidated and the N-terminus of sequence ** was acetylated.

Species	Sequence	pI	Charge at pH 7	GRAVY
Whole affibody	VDNKFNKEMX_1_X_2_X_3_X_4_X_5_EIX_6_X_7_LPNLNX_8_X_9_QX_10_X_11_AFIX_12_SLX_13_DDPSQSANLLAEAKKLNDAQAPK			
α-helix 1	*H_2_N–*VDNKFNKEM–… *	10.08	3	−1.43
α-helix 3	** …–DDPSQSANLLAEAKKLNDAQAPK*–COOH*	3.82	−2	−1.05

The OBOP library was synthesized by solid-phase peptide synthesis (SPPS) on hydroxymethylbenzoic acid (HMBA)-ChemMatrix resin, a crosslinked PEG-based solid support in the form of spherical particles with a diameter range from 100 to 300 μm, and a functional loading of 0.6 mmol/g. ChemMatrix resin is compatible with both organic and aqueous conditions, which are respectively used for the peptide synthesis and for the screening step, and it is, therefore, suitable for library synthesis and selection against target proteins. The ester bond formed between the trimer Gly–Ser–Gly (GSG) spacer and the HMBA linker is stable through the subsequent reactions of peptide elongation and removal of the side chain-protecting group, as well as through the process of library screening. Yet, it undergoes rapid cleavage when exposed to mild alkaline conditions, thereby enabling the release of the peptide from the selected beads; the peptides released in solution can be sequenced by tandem mass spectrometry. Prior work by our group and Albericio et al. demonstrated this approach [37,38,73,74].

## Appendix B. Library Screening and Sequence Identification

Library screening was designed to favor the selection of peptides that bind the constant portion of affibodies (α-helix 3 or homologous regions in α-helices 1 and 2) selectively, that is, in presence of protein impurities produced by the host organisms (e.g., *E. coli* cells). In this work, we resolved to utilize two model targets, namely, an anti-IgG and an anti-HSA affibody, each labeled with a different fluorescent dye, either the green AlexaFluor 488 (AF488) or the red AlexaFluor 594 (AF594). The affibodies were separately labeled with both dies, resulting in two orthogonal target pairs, namely, a green anti-IgG affibody and a red anti-HSA affibody, as well as a red anti-IgG affibody and a green anti-HSA affibody, to decouple potential affibody-dye pairing effect leading to false positives. Thus, to ensure targeting of the constant portion of affibodies, aliquots of the library were incubated with either target pair, and only the beads carrying both colors were chosen. Furthermore, to ensure binding selectivity, the library was screened in competitive conditions, that is, in the presence of *E. coli* host cell proteins (HCP) present in clarified *E. coli* cell lysate.

The library aliquots were initially rinsed with PBS buffer to completely remove any trace of organic solvent, and blocked by incubation with clarified *E. coli* cell lysate at a total protein concentration of 2 mg/mL for 2 h. Affibodies, in fact, are commonly expressed in *E. coli* strains as periplasmically secreted proteins [75]. Thus, to identify affibody-targeting peptides that can be universally applied in either purification or developmental applications, we resolved to screen the library in presence of *E. coli* HCPs. After blocking, the library beads were transferred into a screening mix, comprising either (SM1) AF488-labeled anti-HSA affibody and AF594-labeled anti-IgG affibody in clarified *E. coli* cell lysate, or (SM2) AF594-labeled anti-HSA affibody and AF488-labeled anti-IgG affibody in clarified *E. coli* lysate. The total concentrations of affibody and HCPs were respectively 1 mg/mL and 2 mg/mL in both SM1 and SM2 to reproduce bioprocess-relevant conditions. The library beads were incubated in either SM1 or SM2 overnight, before washing with PBS at pH 7.4 and 0.1% Tween 20 in PBS at pH 6.5, as done in prior work [37,38], to ensure the selection of peptides with high affinity for the affibody product. The washed beads were sorted and individually imaged by fluorescence microscopy. Only the beads carrying both strong red and green fluorescence were selected (Figure A1), whereas beads carrying either a single color or weak fluorescence were discarded together with the fully negative (no fluorescence) ones. The peptides carried by the selected beads were then cleaved from the ChemMatrix surface under alkaline conditions (note: to prevent alkaline degradation of the peptide, the solutions containing the cleaved peptide were immediately neutralized) and sequenced by liquid chromatography coupled with electrospray ionization tandem mass spectrometry (LC–ESI-MS/MS), following a method developed in prior work [37,38] using a ThermoFisher Q Exactive^TM^ High-Field Hybrid Quadrupole-Orbitrap^TM^ Mass Spectrometer coupled to an Easy LC 1200 system with an ESI (electrospray ionization) source. The peptide sequences were obtained by searching the acquired MS data against a peptide database in FASTA format containing all 10^6^ degenerate amino-acid combinations forming the peptide library, using Proteome Discoverer v.1.4 (Thermo Fisher). Among the peptides identified by MS, 16 sequences, listed in Table 2, were selected for their homology in amino-acid composition and sequence, as shown by their “sequence logo display” plot (Figure A2).

The sequences were further analyzed to determine the enrichment factor (f_E_) for each amino acid, which was calculated as the ratio of the frequency at which an amino acid appears in the sequences identified by library screening vs. the probability of that amino acid to be present in a bead randomly picked from the library (Table A2). As expected, owing to the charged nature of the targeted α-helix 1 (+3) and α-helix 3 (−2) regions of affibodies, and the abundance of hydrogen bonding amino acids, the identified peptides were considerably enriched in positively charged (f_E_ = +3.3 for His, Arg, and Lys), negatively charged (f_E_ = +2.5 for Asp), and neutral hydrogen bonding (f_E_ = +3.5) residues; analogously, they were found to be depleted in aromatic amino acids (f_E_ = −0.4). It is interesting to notice that aspartic acid, a negatively charged amino acid, was also enriched, yet it was always flanking positively charged amino acids, as in the triplets DIR, DIH, and DHH. This combination was shown by both experimental binding studies in competitive conditions (Section 2.3) and in silico docking studies (Section 2.5) to denote high binding specificity.

**Table A2:** Factor of amino-acid enrichment (f_E_) produced by the library screening, calculated as the ratio between the frequency of a group of amino acids in the identified sequences and the probability of finding it in a random peptide in the library.

Amino Acid	Probability	Frequency	*f_E_*
Aliphatic (Ala, Ile, Gly)	30%	81%	2.7
Aromatic (Tyr)	10%	6%	−0.4
Neutral hydrophilic	20%	69%	3.5
(Gln, Ser) + Charged	30%	100%	3.3
(His, Lys, Arg) − Charged (Asp)	10%	25%	2.5

## Appendix C. Affibody Binding Capacity vs. Ligand Density

As shown in Section 2.4, the peptide-based adsorbents featured a higher maximum binding capacity (Q_max_) compared to the corresponding positive control adsorbents that employed the proteins targeted by the affibodies as ligands. This can be explained in terms of ligand size and distribution of the affibody proteins on the adsorbent surface at the equilibrium. Peptide ligands, in fact, are considerably smaller (~1 kDa) than both the binding proteins (IgG is ~150 kDa, HSA is ~66.5 kDa, and ErbB2 is ~68.6 kDa) and the target affibodies (~6.5 kDa). This enables a significantly higher density of peptide ligands than proteins on the pore surface of Toyopearl resin. As a result, on peptide–Toyopearl adsorbents, the amount of bound affibodies depends on the size of affibodies (Figure A3A), while, on the positive control adsorbents, it depends on the size and surface density of proteins (Figure A3B).

To evaluate this hypothesis, we prepared three IGKQRI–GSG–Toyopearl adsorbents at different ligand density, namely, 0.045 meq of peptide per mL of resin, 0.11 meq/mL, and 0.26 meq/mL, as well as three HSA–Toyopearl adsorbents at different albumin density, namely, 0.9 mg/mL (1.35 × 10^−5^ meq/mL), 10.5 mg/mL (1.58 × 10^−4^ meq/mL), and 24.6 mg/mL (3.7 × 10^−5^ meq/mL). Every adsorbent was incubated in a solution of anti-HSA affibody at 1 mg/mL in PBS, pH 7.4, for 2 h, and the equilibrium binding capacity (Q_max_) was determined by mass balance based on the final HSA concentration in solution. The values of Q_max_ were finally plotted against the binder density, with either IGKQRI peptide or HSA, as shown in Figure A4. The values of capacity of HSA–Toyopearl resins were found to depend linearly on the density of HSA conjugated to the resin, whereas those of IGKQRI–GSG–Toyopearl resin were found to depend only marginally on the surface density of peptide ligands. These findings corroborate the hypothesis that the size of ligands (and, thus, their density on the chromatographic substrate) is a key controlling factor of binding capacity, perhaps more so than their affinity for the target affibody.
